# Mechanical factors tune the sensitivity of *mdx* muscle to eccentric strength loss and its protection by antioxidant and calcium modulators

**DOI:** 10.1186/s13395-020-0221-2

**Published:** 2020-02-01

**Authors:** Angus Lindsay, Cory W. Baumann, Robyn T. Rebbeck, Samantha L. Yuen, William M. Southern, James S. Hodges, Razvan L. Cornea, David D. Thomas, James M. Ervasti, Dawn A. Lowe

**Affiliations:** 1 Division of Rehabilitation Science and Division of Physical Therapy, Department of Rehabilitation Medicine, University of Minnesota, MMC 388, 420 Delaware Street SE, Minneapolis, 55455 USA; 2 Department of Biochemistry, Molecular Biology and Biophysics, University of Minnesota, 6-155 Jackson Hall, 321 Church Street SE, Minneapolis, 55455 USA; 3 Institute for Physical Activity and Nutrition (IPAN), School of Exercise and Nutrition Sciences, Deakin University, Geelong, VIC 3220 Australia; 4 Division of Biostatistics, University of Minnesota, A460 Mayo Building, 420 Delaware Street SE, Minneapolis, 55455 USA

**Keywords:** Dystrophin, Eccentric contraction, Force drop, Muscle damage, Oxidative stress, Ryanodine receptor, SERCA, Skeletal muscle

## Abstract

**Background:**

Dystrophin deficiency sensitizes skeletal muscle of mice to eccentric contraction (ECC)-induced strength loss. ECC protocols distinguish dystrophin-deficient from healthy, wild type muscle, and test the efficacy of therapeutics for Duchenne muscular dystrophy (DMD). However, given the large lab-to-lab variability in ECC-induced strength loss of dystrophin-deficient mouse skeletal muscle (10–95%), mechanical factors of the contraction likely impact the degree of loss. Therefore, the purpose of this study was to evaluate the extent to which mechanical variables impact sensitivity of dystrophin-deficient mouse skeletal muscle to ECC.

**Methods:**

We completed ex vivo and in vivo muscle preparations of the dystrophin-deficient *mdx* mouse and designed ECC protocols within physiological ranges of contractile parameters (length change, velocity, contraction duration, and stimulation frequencies). To determine whether these contractile parameters affected known factors associated with ECC-induced strength loss, we measured sarcolemmal damage after ECC as well as strength loss in the presence of the antioxidant N-acetylcysteine (NAC) and small molecule calcium modulators that increase SERCA activity (DS-11966966 and CDN1163) or lower calcium leak from the ryanodine receptor (Chloroxine and Myricetin).

**Results:**

The magnitude of length change, work, and stimulation duration ex vivo and in vivo of an ECC were the most important determinants of strength loss in *mdx* muscle. Passive lengthening and submaximal stimulations did not induce strength loss*.* We further showed that sarcolemmal permeability was associated with muscle length change, but it only accounted for a minimal fraction (21%) of the total strength loss (70%). The magnitude of length change also significantly influenced the degree to which NAC and small molecule calcium modulators protected against ECC-induced strength loss.

**Conclusions:**

These results indicate that ECC-induced strength loss of *mdx* skeletal muscle is dependent on the mechanical properties of the contraction and that *mdx* muscle is insensitive to ECC at submaximal stimulation frequencies. Rigorous design of ECC protocols is critical for effective use of strength loss as a readout in evaluating potential therapeutics for muscular dystrophy.

## Introduction

Mutation in the *DMD* gene can detrimentally affect the expression and function of its product dystrophin [1], a protein that stabilizes the sarcolemma during contraction by linking the extracellular matrix to the intracellular cytoskeleton [2]. Lack of dystrophin renders skeletal muscle susceptible to injury [3], particularly eccentric contraction (ECC)-induced strength loss [4–6]. Such loss of strength in the *mdx* mouse model of Duchenne muscular dystrophy (DMD) is associated with cytosolic calcium influx [7], generation of reactive oxygen species (ROS) [8] and ultimately disruption of the mechanisms responsible for activating, generating, and transmitting force. ECC-induced strength loss in skeletal muscle of *mdx* mice can be partially attenuated by treating with antioxidants [9, 10], increasing sarco-endoplasmic reticulum (SR) calcium transport ATPase (SERCA1a) expression [11] or inhibiting resting-muscle SR calcium leak through ryanodine receptor (RyR1) calcium release channels [12].

Mechanical factors of the ECC that cause strength loss in wild type (WT) skeletal muscle include work performed by the muscle [13], initial length of the muscle, amplitude of the length change [14], and maximal muscle tension [15] during the ECC, with the latter being measured as the ratio of maximal eccentric to isometric force (ECC:ISO). We recently reported that in *mdx* muscle, fiber types and variable expression of utrophin, cytoplasmic actins, and SERCA1—but not ECC:ISO—predict ECC-induced strength loss [16]. Call et al. [17] and Baumann et al. [18] also revealed that loss of sarcolemmal excitability due to depolarization of *mdx* fibers following in vivo ECC is a primary mechanism of strength loss and is distinctly different from WT. Such results suggest that different mechanisms govern the initiation of ECC-induced strength loss in dystrophin-deficient and WT skeletal muscle, yet it remains to be seen whether the mechanical factors of an ECC that influence the extent of strength loss are the same in *mdx* as in WT.

While ECC protocols vary across laboratories in the number of contractions, time between contractions, magnitude of length change, velocity of lengthening, and duration of the contraction (Table 1), they all robustly differentiate *mdx* skeletal muscle from WT [5, 6, 16, 19–34]. However, published force losses in *mdx* extensor digitorum longus (EDL) muscle range from 10 to 95% (Table 1) indicating that protocol variables impact the severity of force loss. Therefore, we investigated the extent to which each mechanical variable of an ECC affects force loss in *mdx* skeletal muscle. We determined that a high stimulation frequency is required to induce loss of strength and that the magnitude of the work, length change, and stimulation duration of an ECC primarily dictate the extent of ECC-induced strength loss in *mdx* muscle. We then utilized this knowledge to show that altering the magnitude of ECC length change can better reveal therapeutic efficacy, here in the case of an antioxidant and calcium modulators.

**Table 1:** ECC protocols in research for testing susceptibility of isolated male *mdx* EDL muscle to force loss

Parameter	Allen [19]	Brooks [20]	Faulkner/Chamberlain [21, 22]	Davies [23]	Duan [24]	Gailly [25]	Lowe/Ervasti [16,26–28]^c^	Lynch [29]^c^	Marechal [6]^c^	Mendell/Janssen [30, 31]	Morley/Head [32]	Sweeney/Barton [5, 33]^c^
Mouse age (converted to nearest month)	2–3	5	1–2^a^ [21] 7–10^a^ [22]	2–3	2–3	3–4	3–6 [16, 27] 3 [26] 6 [28]	2–3	1–16	1–5 [30] 2–3 [31]	1–2 and 6–7	3 and 14 [33] 3 [5]
Bath temperature (°C)	22	25	25	Not specified	30	20	25	25	20	30	22–24	23
Number of contractions	10	1	2–6	5	10	7	10	6	12	10	3	5
Time between contractions (s)	30	n/a	10	300	120	10	180	120	180^d^	120	300	240^Ɛ^
Total length change (%)	30	20–60^b^	30^b^	7.7	10	8	10	5–40^b^	15–17^b^	5–10	15	10
Lengthening velocity (*L*_o_/s)	3.0	2^b^	1^b^	6.6–11.1 mm/s	0.5	1.0	0.5	2.0^b^	1.0^b^	0.5 [30] 0.5^b^ [31]	1 mm/s	0.5
Contraction duration (ms)	100	100–300^e^	300^e^	140	200	90	200	25–200^e^	150–170^e^	200	~ 3750^e^	200
Stimulation Frequency (Hz)	120^f^	~ 130^f^	180^f^	Not specified	150	125	175	Not specified	125	150^f^	100	80 and 120 [33] 80 [5]
Isometric force loss (%)	60–70	10–50	~ 75–90	55	~ 55	> 90	80–90	60–70	38	80–95	~ 8–60^g^	64

Studies are representative and do not encompass all published protocols on whole EDL muscle from *mdx* mice studied ex vivo

Protocols are identified based the principal investigator’s laboratory

All *mdx* mice had a variation of the C57BL/10 background unless indicated otherwise

*ECC* eccentric contraction, *L*_*o*_ optimal muscle length

Contraction duration is the time during the eccentric portion of the ECC

Isometric force loss is either the percent change in isometric tetanic force between the first and last contraction generated during the isometric plateau of the ECC or that from separate maximal isometric tetanic contractions before and then following the ECC protocol

^a^C57BL/6 background

^b^Calculated and reported based on fiber length rather than muscle length

^c^Papers that include data on skeletal muscles in addition to EDL muscle

^d^Time between eccentric contractions followed by 15 min between two sets of 6 ECC

^e^Stimulation duration calculated or estimated from publication

^f^Stimulation frequency of isometric tetanic contractions [stimulation frequency of ECC not specified]

^g^Loss of isometric force is age-dependent

## Materials and methods

### Experimental mice

Three-month-old male *mdx* mice (C57BL/10ScSn-DMD*mdx*/J) were generated using founders purchased from Jackson Laboratory (Bar Harbor, ME, USA). All mice were housed in groups of 3–4 per cage on a 14/10-h light/dark cycle with food and water provided ad libitum.

### Study design

Ex vivo ECC protocols were designed to determine which mechanical factors were most influential in initiating ECC-induced force loss. To determine the stimulation frequencies required to manipulate ECC:ISO, we first completed a force-frequency analysis of isolated EDL muscle. Because ECC:ISO was determined to be the dominant factor initiating ECC-induced force loss in WT skeletal muscle (19), we manipulated ECC:ISO by using a passive lengthening (no stimulation, 0 Hz), a stimulation frequency that elicited force half-way between twitch and maximal tetanic forces, and a stimulation frequency that elicited maximal tetanic force. Each stimulation frequency (0, 35, 120 Hz), muscle length change (5, 10, 20, and 30%, L_o_) and contraction velocity (0.125, 0.25, 0.5, 1.0, 2.0, and 3.0 L_o_/s) were tested for a total of 51 ECC protocols (Additional file 1: Figure S1). Each protocol was designed based on those tested in WT muscle [15], keeping within physiological limitations and using the dual-mode lever system (300B-LR; Aurora Scientific Inc., Aurora, ON, Canada). The study’s primary outcome was change in maximal isometric force and changes in maximal tetanic rates of contraction and relaxation following ECC. To prevent a metabolic influence on the ECC protocol, we only used 10 ECC separated by three min.

To determine which mechanical factors contribute to torque loss of dystrophin-deficient skeletal muscle in vivo, we completed ECC of the anterior crural muscles (tibialis anterior, EDL and extensor hallucis longus) where ECC:ISO (0.52–2.37), degree of ankle rotation (0–40°), contraction velocity (0–2000°/s), and contraction duration (0–320 ms) were manipulated in *mdx* mice. Lastly, we tested the effect of varying ECC mechanical factors on the level of protection against strength loss by ROS and calcium modulators in isolated EDL muscle using N-acetylcysteine (NAC) and small molecule modulators of SERCA1a and RyR1, respectively. In these experiments, we measured rates of relaxation and contraction, in addition to strength loss, in order to gain insight on how the calcium modulators impact physiological outcomes related to fiber calcium kinetics.

### Ex vivo muscle preparation

Mice were anesthetized with sodium pentobarbital (75 mg/kg body mass). EDL muscles (15.10 ± 0.12 mg; 13.36 ± 0.04 mm; *n* = 208) were removed and mounted on a dual-mode muscle lever system (300B-LR; Aurora Scientific Inc.) with 5–0 suture in a 1.2 mL bath assembly filled with oxygenated (95:5% O_2_:CO_2_) Krebs-Ringer bicarbonate buffer maintained at 25 °C. Muscles were adjusted to their anatomical optimal length (L_o_) based on resting tension [35]. Muscles remained quiescent in the bath for 5 min before performing maximal isometric tetanic contractions every 2 min. The muscle was stimulated to contract using maximal voltage (150 V) for 200 ms at 175 Hz until force plateaued within 5 mN from one contraction to the next (381 ± 4 mN; 15.80 ± 0.16 N/cm^2^). Maximal rate of tetanic contraction (+ dP/dt) and relaxation (− dP/dt) were calculated from maximal isometric tetanic force (*P*_o_) and muscle length was measured from myotendinous junction to myotendinous junction using digital calipers.

### Force-frequency analysis

Two minutes following plateau of isometric force, a force-frequency analysis was completed. EDL muscles completed 10 isometric contractions (10, 20, 25, 30, 40, 50, 60, 80, 120, 160 Hz) with a 3-min rest between each. Four muscles were used for the force-frequency protocol; they were not used for subsequent ECC protocols.

### ECC protocol

Two minutes following plateau of isometric force in separate cohorts of mice, a series of 10 ECC were performed (Additional file 1: Figure S1). For each ECC, the muscle was passively shortened 50% of the total length change and then stimulated while the muscle was simultaneously lengthened at a given velocity (see Additional file 1: Figure S1 for specific parameters). Work was calculated from the total force integrated over length change during the first ECC contraction at 120 Hz. Immediately following the 10th ECC, the muscle was readjusted to *L*_o_ and *P*_o_, + dP/dt and – dP/dt were re-measured.

### ECC protocol for NAC and calcium flux modulators

Following plateau of isometric force in separate cohorts of *mdx* mice, EDL muscles (15.95 ± 0.17 mg; 13.66 ± 0.04 mm; 395 ± 7 mN; 15.91 ± 0.28 N/cm^2^; *n* = 125) were incubated with NAC (20 mM with the addition of 1% DMSO) or varying concentrations of small molecule SERCA1a activators (DS-11966966 and CDN1163—0.1, 1.0, 10, or 100 μM dissolved in dimethylsulfoxide (DMSO)), small-molecule inhibitors of resting RyR1 leak (Chloroxine and Myricetin—0.01, 0.1, 1.0, 10, or 100 μM dissolved in DMSO), or a combination of molecules. DMSO did not change *P*_o_ of EDL muscles compared to *P*_o_ measured during non-DMSO experiments (386 ± 16 vs. 381 ± 4 mN; *p* = 0.732). After 30 min of incubation where addition of NAC and/or calcium modulator drug also did not affect isometric force production (*p* ≤ 0.675), *P*_o_ and + dP/dt and − dP/dt were measured before a series of 10 ECC. For these ECCs, the muscle was passively shortened to 97.5% *L*_o_ and then stimulated while the muscle was simultaneously lengthened to 102.5% *L*_o_, corresponding to a 5% total length change (done at 0.5 *L*_o_/s, 200 ms duration). Immediately following the 10th ECC, the muscle was readjusted to *L*_o_ and *P*_o_ was measured.

### In vivo mouse preparation

Mice were anesthetized with isoflurane and maximal isometric torque (2.84 ± 0.06 mN m; 83.6 ± 2.0 mN m/kg; *n* = 83) of the anterior crural muscles was measured as previously described [20]. Torque-frequency relationship was then established at varying stimulation frequencies (20, 40, 60, 80, 100, 125, 150, and 200 Hz) with a 45-s rest between each contraction. Eight mice were used for the torque frequency protocol; they were not used for subsequent ECC protocols.

### ECC protocol in vivo

One minute after maximal torque was measured, anterior crural muscles were injured by performing 70 electrically stimulated ECC. Stimulation frequencies of 0, 52, 71, 93, and 150 Hz were used to manipulate ECC:ISO, corresponding to passive lengthening or frequencies required to produce 0, 50, 75, 90, or 100% torque between a twitch and tetanus, respectively. Degree of ankle rotation (0, 5, 10, 20, and 40°), contraction velocity (0, 62, 125, 250, 500, 1000, and 2000°/s), and contraction duration (2.5, 5, 10, 20, 40, 80, 160, and 320 ms) were also manipulated to generate a total of 20 protocols. Each ECC was separated by 10 s. Work was calculated from total torque integrated over length change during the first ECC contraction at 150 Hz. Five minutes following the last ECC, isometric tetanic torque was measured.

### Evan’s blue dye (EBD) assay

EBD was diluted in PBS to 5 mg/mL, filter sterilized with a 0.2 μm filter and injected intraperitoneal at 100 μL/10 g body mass 24 h before 15 ECC, as previously described [9]. Fifteen ECC were chosen because it optimally separated torque loss between testing protocols. Twenty-four hours following ECC-induced injury, tibialis anterior muscles were removed, cryopreserved, and later sectioned and stained. Images were acquired on a Leica DM5500 B microscope equipped with a Leica HC PLAN APO × 10 objective and stitched together with LASX software (Leica) to allow visualization of the entire tibialis anterior. MyoVision software (https://www.uky.edu/chs/muscle/myovision) was used to determine the percentage of EBD-positive fibers in whole tibialis anterior images.

### Statistics

Prism 7 software (GraphPad, San Diego, CA) was used for all statistical analyses except those reported in Table 2, which were computed using JMP (v. 13.1.0 Pro, SAS Institute Inc., Cary NC). For 0 and 35 Hz ex vivo ECC protocols, one-way ANOVA was used to test differences between ECC groups for muscle tension and loss of isometric force compared to initial, and one-way ANOVA with Bonferronized post-hoc tests were used to analyze muscle tension and loss of isometric force when collapsed into length change groups. For the 120 Hz ex vivo ECC protocols, one-way ANOVA was used to test differences between ECC groups for muscle tension and work while a one-way ANOVA with Bonferronized post-hoc tests was used to calculate muscle tension when collapsed into length changes. For ex vivo loss of isometric force, one-way ANOVA with Bonferronized post-hoc tests were used to analyze differences within each length change. When ECC protocols were collapsed into groups by length change, contraction velocity or contraction duration, one-way ANOVA with Bonferronized post-hoc tests were used. Loss of isometric torque and work in vivo between stimulation frequencies and angle rotation was tested using one-way ANOVA with Bonferronized post-hoc tests. A one-way ANOVA with Bonferronized post-hoc tests were used to analyze Evan’s blue dye uptake in vivo, changes in isometric and eccentric force between groups when NAC and calcium modulators were added to the ex vivo set-up, and for SERCA activity assays.

**Table 2:** Predictors of ECC-induced strength loss in isolated EDL and anterior crural muscles of *mdx* mice

	ECC:ISO	Work	Length/angle change	Contraction velocity	Contraction duration
Ex vivo—(isometric force loss)	0.310 (*p* < 0.001)	0.563 (*p* < 0.001)	0.538 (*p* < 0.001)	0.030 (*p* = 0.084)	0.455 (*p* < 0.001)
In vivo—(isometric torque loss)	0.400 (*p* < 0.001)	0.542 (*p* < 0.001)	0.780 (*p* < 0.001)	0.005 (*p* = 0.606)	0.311 (*p* < 0.001)

*ECC:ISO* ratio of maximal eccentric to maximal isometric force

Values = coefficient of determination (*R*^2^) following 10 and 70 ECC for ex vivo and in vivo contractions, respectively

We compared mechanical factors as single predictors of force loss ex vivo and torque loss in vivo using *R*^2^ for the logarithm of force/torque loss (percent of initial) regressed on each mechanical factor individually. Logarithmic transformation was chosen due to the large variation between and within ECC protocols in loss of isometric force/torque.

Data are presented as mean ± SEM with significance set at *p* < 0.05.

## Results

### Magnitude of length change and duration of ECC best predict sensitivity to ECC-induced force loss in isolated mdx EDL muscle

**Fig. 1:**
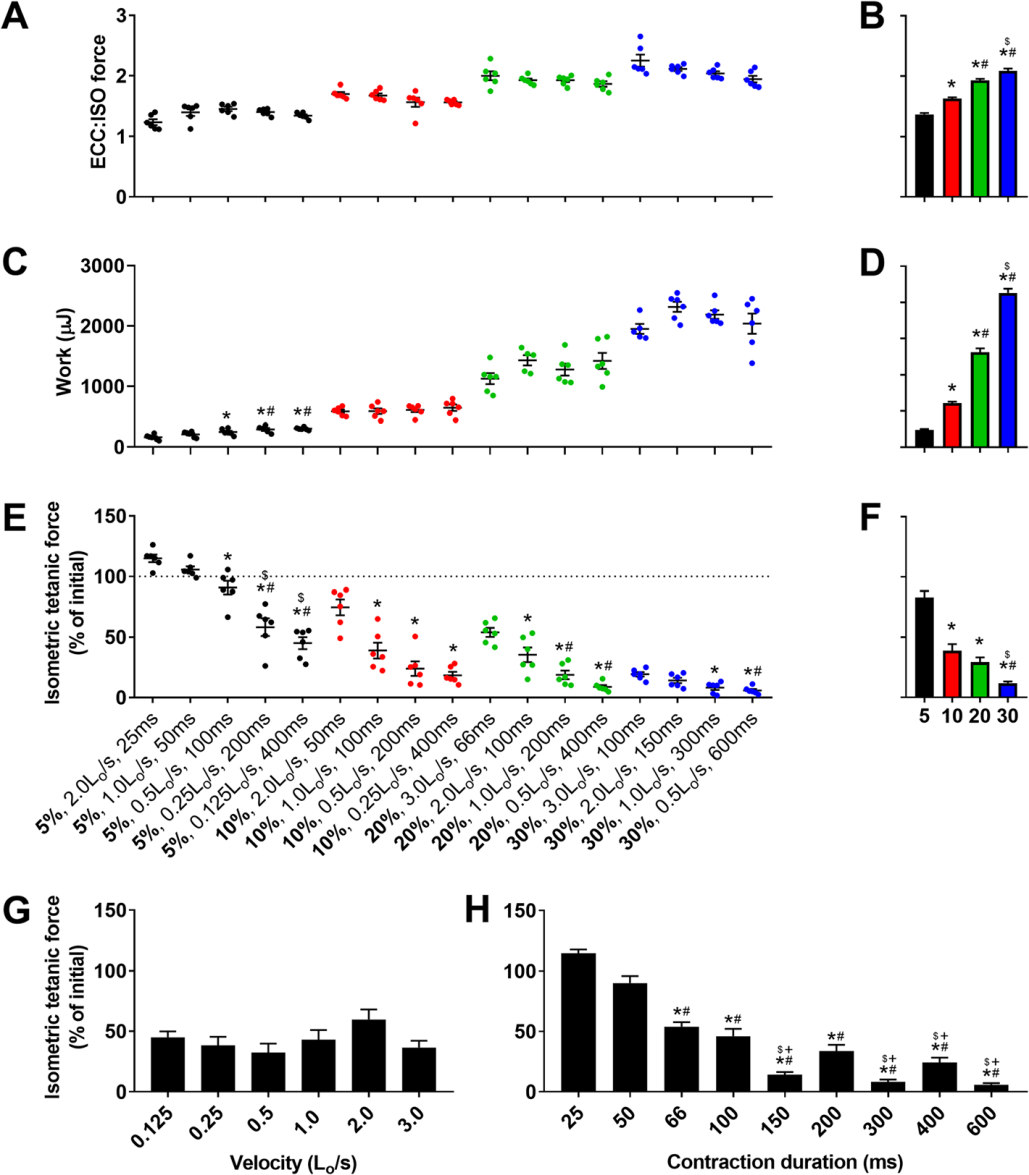
Mechanical factors that impact the sensitivity of *mdx* EDL muscle to ECC ex vivo. **a** Maximal eccentric force as a fraction of maximal isometric tetanic force for each eccentric protocol and **b** when grouped into length changes. **c** Work completed during the first contraction of each eccentric protocol and **d** when grouped into length change. Different from *5%, ^#^10%, and ^$^20%. **e** Maximal isometric tetanic force (120 Hz) following 10 eccentric contractions as a percent of maximal isometric force before ECC (initial). Different from the *first, ^#^second, and ^$^third protocol within a given length change. **f** Isometric tetanic force as a percent of initial for each protocol when collapsed into length changes and **g** velocities. Different from *5%, ^#^10%, and ^$^20%. **h** Isometric tetanic force as a percent of initial for each protocol when grouped into contraction durations. *L*_o_, optimal muscle length. Different from *25 ms, ^#^50 ms, ^$^66 ms, and ^+^100 ms. Data are mean ± S.E.M with significance set at *p* < 0.05. *N* = 5–6/protocol

We designed ECC protocols within physiological ranges of contractile parameters including length change, velocity, contraction duration, and stimulation frequencies (Additional file 1: Figure S1). When EDL muscles were maximally stimulated during ECC (120 Hz), force (Fig. 1a, b; *p* < 0.001), and work (Fig. 1c, d; *p* < 0.001) increased as length change increased. Analyzing the effect of contraction velocity and duration within given length changes showed that ECCs with slower velocities and longer durations resulted in greater loss of isometric force compared to fast and short ECCs (Fig. 1e). When ECC protocols were grouped by length change, there was a length change-dependent loss of isometric force (Fig. 1f; *p* < 0.001). Grouping ECC protocols by contraction velocity revealed no effect on loss of isometric force (Fig. 1g; *p* = 0.146), but when grouped by duration of the ECC, longer durations resulted in significantly greater force losses down to 94% loss at 600 ms (Fig. 1h; *p* < 0.001). Regression analyses show that the strongest predictor of strength loss was work completed by the muscle during the first ECC, followed by length change, duration, and then ECC:ISO (Table 2). Velocity of the ECC did not predict force loss. Maximal tetanic rates of contraction and relaxation were measured as additional indices of contractile function affected by ECC. Similar to force loss, for a given length change, slower velocities, and longer contraction durations resulted in greater losses of rates of contraction (Additional file 2: Figure S2A, B) and relaxation (Additional file 2: Figure S2C, D).

To determine if maximal activation of muscle is required, we analyzed the same ECC parameters during submaximal stimulation frequencies of *mdx* EDL muscles (Additional file 3: Figure S3A). Following a passive lengthening protocol (0 Hz), isometric force as a percent of initial isometric force differed between protocols (Additional file 4: Figure S4A; *p* = 0.012); however, none of the lengthening protocols resulted in loss of force (Additional file 4: Figure S4A, B). At a stimulation frequency of 35 Hz eliciting submaximal force, ECC:ISO differed between protocols (Additional file 5: Figure S5A; *p* < 0.001) with 30% lengthening generating 72% greater eccentric than isometric force (Additional file 5: Figure S5B; *p* < 0.001). Similar to the passive lengthening protocol, following the 10th ECC of each, there was a difference in isometric force as a percent of initial between protocols (Additional file 5: Figure S5C; *p* < 0.001). When submaximal ECC protocols were assessed by length change, only the 30% group lost isometric force (Additional file 5: Figure S5C, D; *p* = 0.029) and the loss was minimal (6%) despite the large eccentric force that was generated. Together, these data show that the factors of ECC making *mdx* EDL muscle sensitive to force loss are the magnitude of lengthening during the contraction and the duration of the ECC but only when stimulation is maximal.

### Magnitude of angle change best predicts sensitivity to eccentric torque loss of mdx anterior crural muscles in vivo

**Fig. 2:**
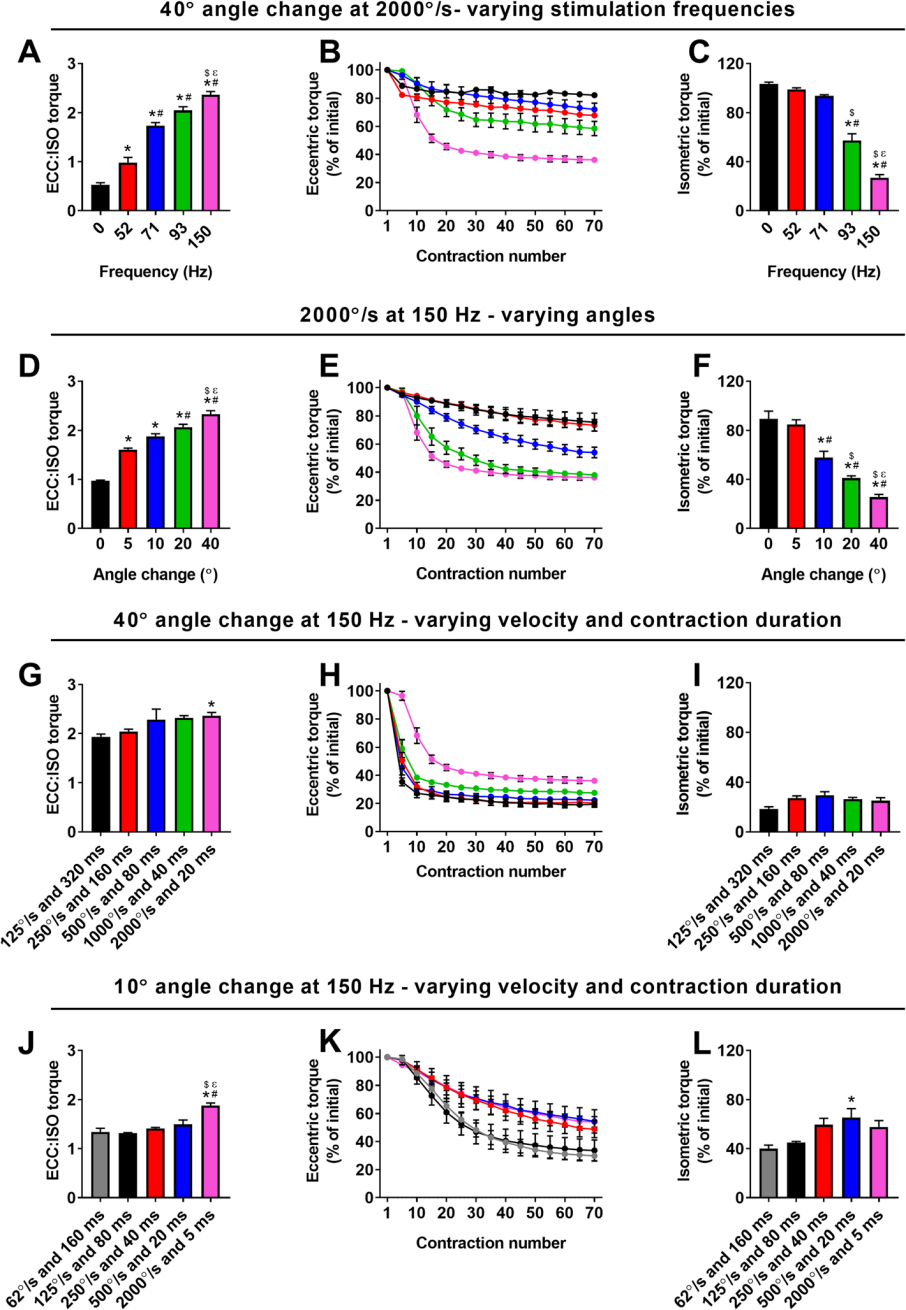
Mechanical factors that impact sensitivity of *mdx* anterior crural muscles to ECC in vivo. **a** Eccentric torque as a ratio of maximal isometric torque, **b** eccentric torque loss, and **c** isometric torque as a percent of initial following 70 eccentric contractions at varying stimulation frequencies muscles using a 40° angle change at 2000°/s. Different from ^*^0 Hz, ^#^52 Hz, ^$^71 Hz, and ^Ɛ^93 Hz. **d** Eccentric torque as a ratio of maximal isometric torque using, **e** eccentric torque loss, and **f** isometric torque as a percent of initial following 70 eccentric contractions at varying degrees of angle change at 2000°/s and 150 Hz. Different from ^*^0°, ^#^5°, ^$^10°, and ^Ɛ^20°. **g** Eccentric torque as a ratio of maximal isometric torque, **h** eccentric torque loss, and **i** isometric torque as a percent of initial following 70 eccentric contractions at varying velocities and contraction durations a using a 40° angle change. Different from ^*^125°/s. **j** Eccentric torque as a ratio of maximal isometric torque, **k** eccentric torque loss, and **l** isometric torque as a percent of initial following 70 eccentric contractions using a 10° angle change at varying velocities and contraction durations. Different from ^*^62°/s, ^#^125°/s, ^$^250°/s, and ^Ɛ^500°/s. Data are mean ± S.E.M with significance set at *p* < 0.05. *N* = 3–9/protocol

To test if the mechanical factors that best predicted force loss ex vivo also had an impact in vivo, we measured strength loss of *mdx* anterior crural muscles during and following 70 ECC. ECC produced in vivo have similar properties as those ex vivo with the exception that muscle lengthening occurs by rotation about a joint, here ankle plantarflexion, reported as angle change with velocity in degree/s and strength being measured as torque. As expected, isometric and ECC:ISO torques increased with increasing stimulation frequency (Additional file 3: Figure S3B and Fig. 2a), and there was loss of isometric torque following 70 ECCs only at the highest frequencies (Fig. 2b, c; *p* < 0.001). Because length change was a strong predictor of isometric force loss in isolated EDL muscle (Table 2), we then manipulated ECC:ISO by changing the degree of ankle rotation, the in vivo equivalent of muscle length change. ECC:ISO of the anterior crural muscles increased with angle change (Fig. 2d; *p* < 0.001) which resulted in a greater loss of eccentric and isometric torque following 70 ECCs (Fig. 2e, f; *p* < 0.001).

Contraction velocity and duration were manipulated next. Each condition elicited high ECC:ISO torque with only the fastest velocity and shortest duration being different (Fig. 2g; *p* = 0.022). Interestingly, this slightly higher ECC:ISO combination resulted in substantially less eccentric torque loss (Fig. 2h; *p* < 0.001) but no difference in loss of isometric torque (Fig. 2i; *p* = 0.075). When the angle change was reduced to 10°, relatively low ECC:ISO torques yielded similar torque losses (Fig. 2j–l; *p* < 0.001). Because work was the strongest predictor of isometric force loss in isolated EDL muscle (Table 2), we measured work in all in vivo protocols at 150 Hz and determined a greater change in ankle rotation resulted in more work (Additional file 6: Figure S6A, B; *p* < 0.001). Overall, the data indicate that the degree of ankle rotation, and therefore, the change in muscle length, tunes the sensitivity of *mdx* anterior crural muscles to ECC in vivo. This conclusion was substantiated by regression analyses showing that ECC-induced torque loss was strongly predicted by angle/length change (Table 2). Work completed during the first ECC, followed by stimulation duration and ECC:ISO also significantly predicted torque loss while contraction velocity did not. These data are similar to the ex vivo results except that work was the strongest predictor in isolated EDL muscle.

### Sarcolemmal damage is associated with the muscle length change of an eccentric contraction in vivo

**Fig. 3:**
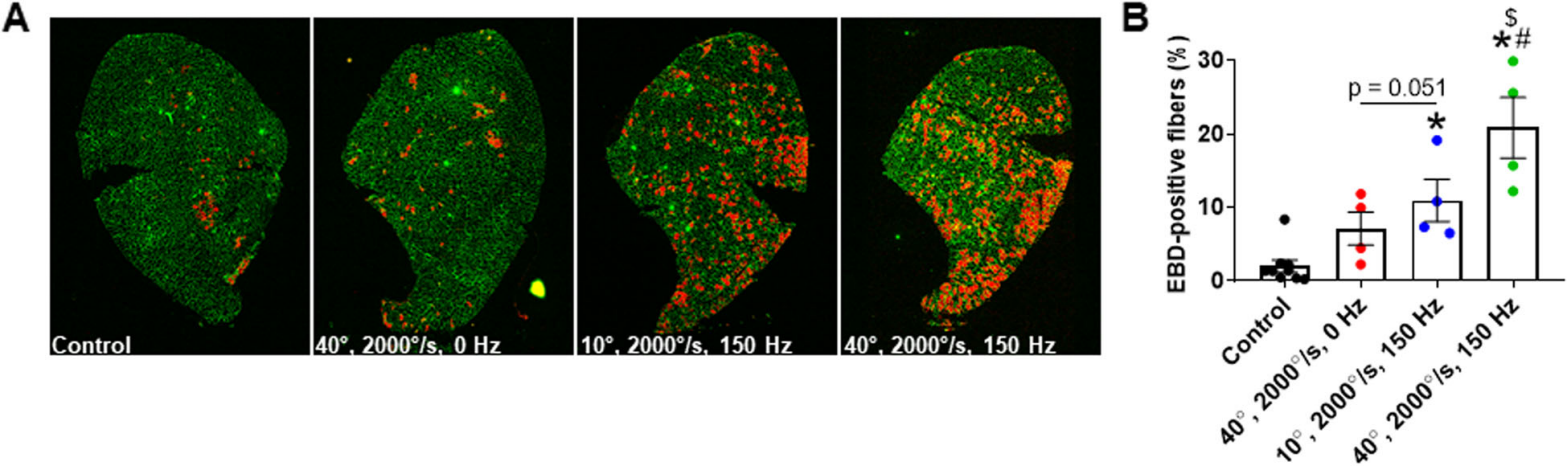
Mechanical factors of an ECC impact sarcolemmal permeability of *mdx* anterior crural muscles in vivo. **a** Fluorescent microscopy for Evan’s blue dye (EBD; red) and laminin (green) of *mdx* tibialis anterior muscle exposed to three eccentric contraction protocols that vary by angle change (10° and 40°) and stimulation frequency (0 and 150 Hz). **b** Quantification of the percentage of EBD-positive fibers in *mdx* tibialis anterior muscle subjected to one of three eccentric contraction protocols. No ECC = contralateral tibialis anterior not subjected to eccentric contractions, ECC = subjected to eccentric contractions. ^*^Different from control; ^#^ 40°, 2000°/s, 0 Hz; ^$^10°, 2000°/s, 150 Hz. Data are mean ± S.E.M with significance set at *p* < 0.05. *N* = 4/protocol

Sarcolemmal damage of *mdx* skeletal muscle positively correlates with ECC ex vivo [5] and in vivo [9]. To determine whether the level of muscle damage is associated with mechanical factors of an ECC, we measured EBD uptake in *mdx* tibialis anterior muscles exposed to three ECC protocols in vivo that varied by angle change and stimulation frequency. There was an angle change-dependent increase in EBD-positive fibers while passive rotation did not differ in EBD-positive fibers from the contralateral muscle (Fig. 3). These data indicate that the magnitude of ankle rotation and therefore, the change in tibialis anterior muscle length impacts sarcolemmal damage in *mdx* muscle exposed to ECC resulting in up to 21% of the fibers being positive for EBD.

### The antioxidant NAC protects mdx muscle from ECC-induced force loss in a muscle length change-dependent manner

**Fig. 4:**
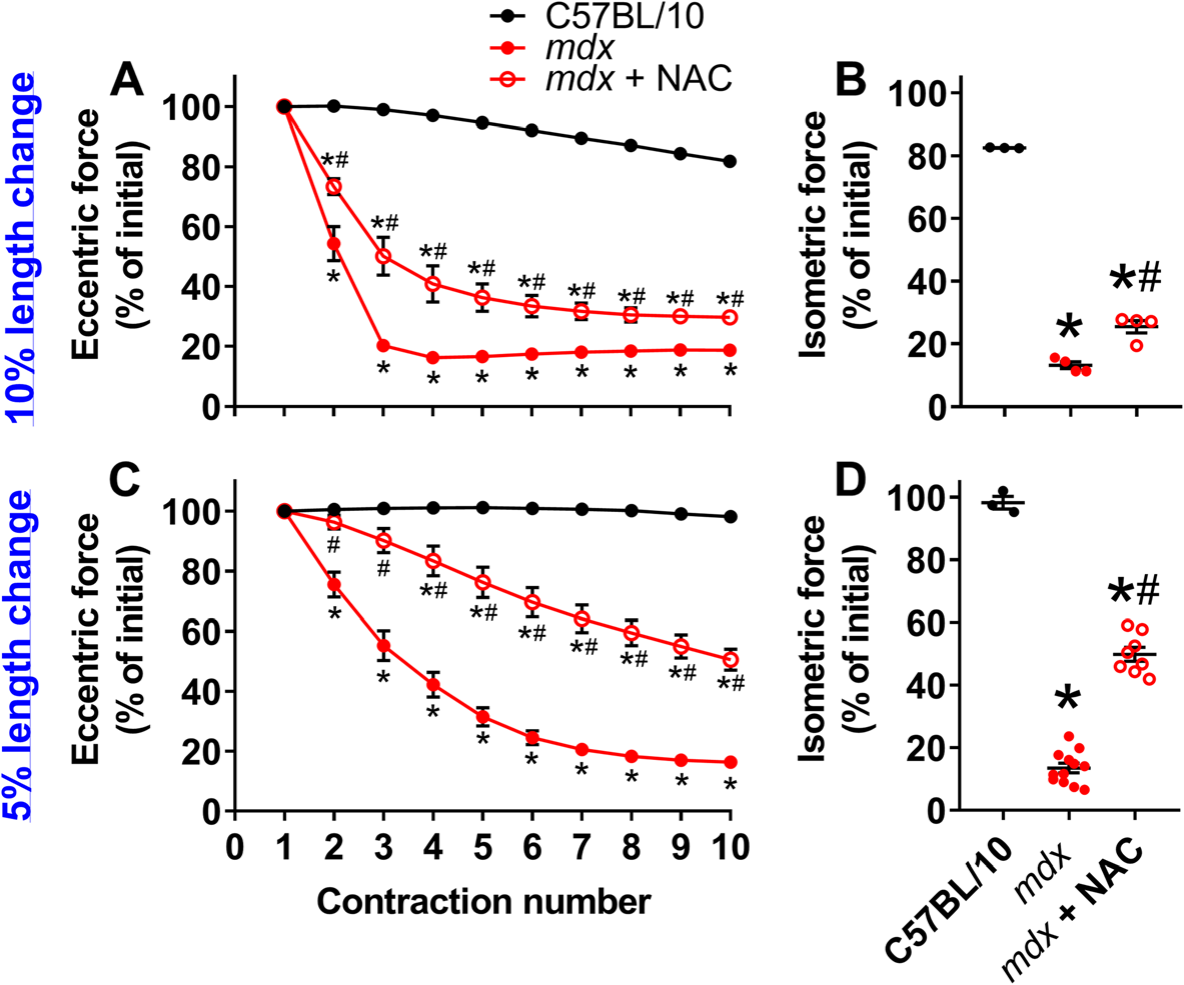
Magnitude of ECC length change differentially affect the impacts of NAC on *mdx* EDL muscle. **a** Eccentric and **b** isometric force losses as percentages of initial forces in isolated EDL muscle of C57BL/10, *mdx* and *mdx* incubated with 20 mM NAC challenged by ECCs with a 10% length change or **c**, **d** 5% length change. ^*^Different from C57BL/10, ^#^different from *mdx*. Data are mean ± S.E.M with significance set at *p* < 0.05. *N* = 3–12/protocol or group

ECC-induced force loss of isolated *mdx* EDL muscle is associated with oxidative stress [9, 36]. We have previously shown that addition of NAC partially protects *mdx* EDL muscle from losing force from ECCs of a 10% length change [9], and here we confirmed this result (Fig. 4a, b). Because length change strongly tunes the sensitivity of *mdx* EDL muscle to ECC (Fig. 1), we posited that modulations in force loss caused by varying the length change would be related to oxidative stress. When length change was reduced to 5%, the force loss between ECC 2–8 was reduced relative to that at 10%, and the addition of NAC provided more substantial protection (Fig. 4c, d) compared to 10% length change (Fig. 4a, b). These data indicate that the magnitude of length change of an ECC impacts the protection from ECC-induced force loss afforded by NAC.

### Small-molecule calcium modulators protect mdx muscle from ECC-induced force loss in a length change-dependent manner

**Fig. 5:**
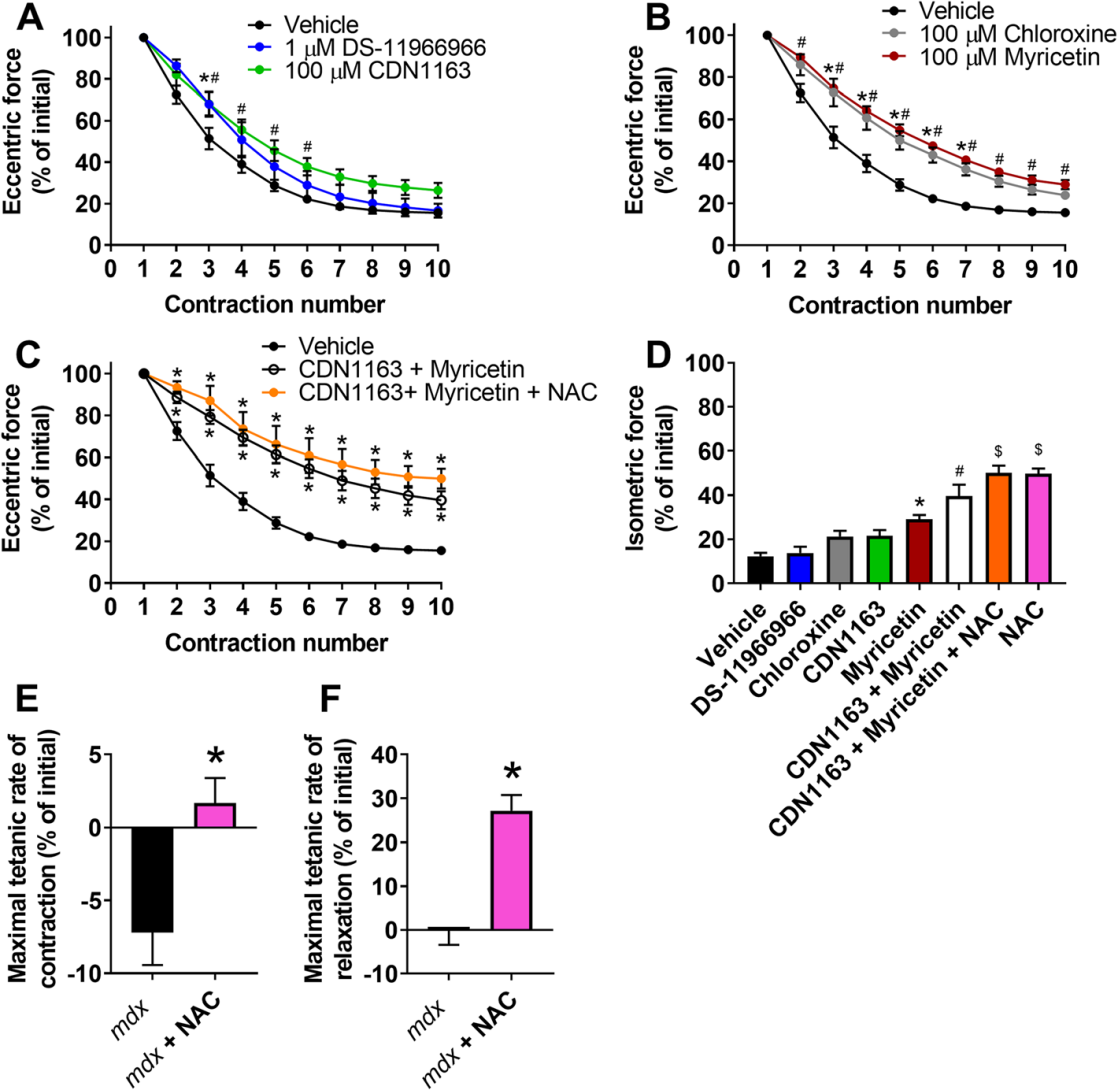
Activation of SERCA1a and inhibition of RyR1 leak attenuates ECC-induced force loss of *mdx* muscle. **a** Eccentric force loss of isolated *mdx* EDL muscle incubated with small molecule SERCA1a activators (DS-11966966 and CDN1163) at their optimal concentration (^*^DS-11966966 different from vehicle and ^#^CDN1163 different from vehicle); **b** ryanodine receptor (RyR1) leak inhibitors (Chloroxine and Myricetin) at their optimal concentration (^*^Chloroxine different from vehicle, ^#^Myricetin different from vehicle); **c** a combination of CDN1163 and Myricetin, a combination of CDN1163 + Myricetin + 20 mM N-acetyl cysteine (NAC) (^*^different from vehicle); and (**d**) isometric force as percent of initial immediately following the 10th eccentric contraction with and without all SERCA1a and RyR1 small-molecule modulators. **e** Maximal rates of tetanic contraction and **f** maximal rates of tetanic relaxation as a percent of initial of *mdx* EDL muscle with or without 20 mM N-acetyl cysteine (NAC). ^*^Different from *mdx*. All ECC protocols were completed with a 5% length change. ^*^Different from vehicle, ^#^different from vehicle and DS-11966966, and ^$^different from vehicle, DS-11966966, Chloroxine, CDN1163, and Myricetin. Data are mean ± S.E.M with significance set at *p* < 0.05. N = 5–12/compound or combination of compounds

Changes in cytosolic calcium have been implicated in ECC-induced force loss of *mdx* muscle [11, 12, 37], and increasing SERCA1a activity or reducing RyR1 SR leak have been shown to ameliorate several dystrophic phenotypes [12, 37–40]. Here, we incubated *mdx* EDL muscles with small-molecule calcium modulators that were previously identified through high-throughput screening assays as activators of SERCA (DS-11966966 and CDN1163; Additional file 7: Figure S7, [41–44]) or inhibitors of RyR1 leak (Chloroxine and Myricetin, [45]). After demonstrating that these calcium modulators affected contraction kinetics, particularly relaxation (Additional file 8 Figure S8 and Additional file 9: Figure S9), we tested four concentrations of each modulator (Additional file 10: Figure S10) and measured significant attenuation of ECC-induced force loss (Fig. 5a, b). The effects of the best performing SERCA1a activator (CDN1163) and RyR1 inhibitor (Myricetin) were additive in providing greater protection against ECC-induced force loss than either agent alone (Fig. 5c, d). To further examine the effect of modulating both calcium and oxidative stress, we incubated *mdx* EDL muscle with CDN1163 + Myricetin + NAC and measured an even greater protection from ECC-induced force loss sparing ~ 50% of ECC force at contraction 10 (Fig. 5d). However, isometric force following the 10th contraction was not different between CDN1163 + Myricetin + NAC and NAC alone (Fig. 5d). Because NAC provided the greatest protection from strength loss, we measured maximal rates of contraction and relaxation and determined that NAC improves both (Fig. 5e, f), suggesting that scavenging of ROS impacts RyR1 and SERCA1a activity in isolated *mdx* EDL muscle.

## Discussion

Sensitivity to ECC has become a hallmark of dystrophin-deficient skeletal muscle in animal models of DMD since its first report [5]. Even though research using DMD mouse models routinely utilizes ECC as a robust phenotype to test the efficacy of genetic and pharmacological interventions, the reason why this specific type of contraction causes strength loss is not clear. Here, we aimed to identify which mechanical component of an ECC initiates ECC-induced strength loss in *mdx* mice. We found that the magnitude of work, length change, and stimulation duration of an ECC are all strong predictors of strength loss and that a high stimulation frequency to elicit near-maximal strength generation is required. These mechanical factors of an ECC explain why the magnitude of strength loss in *mdx* muscle varies so greatly from laboratory to laboratory (Table 1). We utilized this new knowledge in *mdx* mouse muscle to optimize parameters of our ECC protocol in order to effect significantly greater protection against force loss by a ROS scavenger and small-molecule calcium modulators.

The magnitude of muscle length change ex vivo*,* or degree of ankle rotation in vivo, were dominant factors sensitizing *mdx* muscle to ECC-induced strength loss, indicating that a major component of sensitivity is associated with the degree of stretch imposed on the muscle during contraction, and this is similar to WT muscle [14, 46]. The amount of work completed during the first ECC, which is directly proportional to length change, was an equally strong predictor of ECC-induced strength loss and similar to previous reports in WT muscle as well [13]. The duration of the contraction was also associated with, and predictive of, the sensitivity of *mdx* muscle to ECC (Table 2). Interestingly, ECC:ISO were less predictive and contraction velocity was not predictive, which differs from dystrophin-containing WT muscle [15, 47, 48]. Collectively, mechanical factors of a contraction impact the sensitivity of *mdx* muscle to ECC similarly in ex vivo and in vivo muscle preparations.

There are multiple ECC protocols used to differentiate WT skeletal muscle from dystrophin-deficient skeletal muscle, as well as testing the efficacy of various genetic therapies and pharmacological interventions for DMD (e.g., Table 1). With such disparity in total length change (8–60% of the muscle length), velocity of the lengthening contraction (0.25–3 *L*_o_/s), contraction duration (90–3750 ms) and stimulation frequency (80–180 Hz) among protocols, it was unknown which mechanical factors of the ECC contributed to the varying degrees of force loss measured in isolated EDL muscle of *mdx* mice (10–95%). Our study now provides the first evidence that multiple factors of the ECC impact the degree of force loss in *mdx* muscle ex vivo and in vivo; however, our data does not completely describe the differences in force loss between protocols. For example, the Gailly ECC protocol [25] induced greater than 90% force loss but with only an 8% length change, 90 ms contraction duration and 125 Hz stimulation. In contrast, the Marechal ECC protocol [6] only induced a 38% loss of force but with a 15–17% length change, 100 ms contraction duration and 125 Hz stimulation. Therefore, while mechanical factors of an ECC may determine the loss of force in *mdx* EDL muscle, environmental factors such as bath temperature, number of contractions, and time between contractions may also impact sensitivity and should be controlled accordingly.

A novel finding of this study is that dystrophin-deficient skeletal muscle was only sensitive to ECC when near-maximal muscle fiber activation was achieved through a high stimulation frequency. There was no loss of strength when *mdx* muscle was mechanically lengthened without stimulation (i.e., passively stretched within anatomical limits) (Additional file 4: Figure S4 and 2C) and when submaximal stimulation frequencies were used for ECCs ex vivo or in vivo, regardless of the eccentric force or torque generated, minimal strength was lost (Additional file 5: Figure S5 and 2C). This finding could be interpreted as dystrophin-deficient skeletal muscle actually being quite resilient to ECC, as maximal muscle activation rarely occurs with voluntary movements in vivo. It is important to keep in mind that skeletal muscle of *mdx* mice does not completely recapitulate the human DMD phenotype, particularly with regard to pathology, and may contribute to the need of maximal ECC to induce strength loss. Thus, while submaximal stimulation may not induce force or torque loss in *mdx* mice, submaximal ECCs may affect patients with dystrophin deficiency. To our knowledge, there have not been clinical investigations that have subjected patients with DMD to ECC. Our results may offer researchers a foundation for examining exercise paradigms that include both submaximal concentric and eccentric contractions in patients with DMD.

ECC strength loss of *mdx* muscle was originally thought to be caused by muscle damage [5], which would agree with our new data indicating that mechanical parameters of an ECC impact the extent of sarcolemmal permeability (Fig. 3). However, there is a well-documented disconnect [5] between the amount of damage and force loss following ECCs in *mdx* muscle, as is seen in this study with damage measured by EBD-positive fibers (21%) not matching with the degree of total strength loss (70%). EBD staining in dystrophin-deficient mouse muscle associates with IgG, IgM, and albumin staining [49] further indicating sarcolemmal damage—although the extent that membrane lesions smaller than those allowing infiltration of such molecules contribute to strength loss is not clear. Nonetheless, these results lead us to propose that the majority of strength loss in *mdx* muscle exposed to ECC is not merely the result of damage to the sarcolemma, but rather a complex, multi-factorial insult that involves oxidative stress and cytosolic calcium disruption, which culminates in fiber depolarization and inexcitability [17, 18]. Furthermore, we propose that the various factors involved can be modulated to produce a graded sensitivity of *mdx* muscle to strength loss by (1) altering mechanical parameters of an ECC, such as shortening the ECC length change from 10 to 5% and (2) directly altering calcium kinetics (SERCA1a and RyR1 small molecule modulators) or redox balance within the muscle. This idea is supported by measuring a greater protection with NAC at 5% compared to a 10% length change, which we hypothesize is due to variable levels of mechanically induced oxidative stress and by previous work highlighting that protection from ECC-induced strength loss of *mdx* muscle can be achieved with (a) antioxidants [9, 10], similar to our current results with NAC (Fig. 4); (b) blocking stretch-activated calcium channels [7]; or (c) inhibition of RyR1 leak [12], comparable to our results with Chloroxine and Myricetin (Fig. 5). Adding to the list, ours is the first study to demonstrate the benefit of pharmacologically increasing SERCA activity in protecting *mdx* muscle from ECC strength loss, similar to that accomplished by SERCA1a overexpression [11].

## Conclusions

Our results demonstrate that *mdx* muscle becomes sensitized to ECC based on the magnitude of work, length change, and stimulation duration of the ECC, with a high stimulation frequency also being required. We posit that it is essential, when testing an intervention in dystrophin-deficient skeletal muscle using ECC, that an understanding of the ECC protocol parameters and predicted outcome is judiciously applied. Here, we show how such awareness can be utilized for testing an antioxidant and calcium modulators as potential DMD therapeutics, demonstrating a complex interplay of biological factors underlying strength loss in *mdx* muscle.

## Supplementary information

**Additional file 1: Figure S1.** Summary of ex vivo eccentric contraction protocols used in this study. L_o_ = optimal muscle length.

**Additional file 2: Figure S2.** Rates of contraction and relaxation of *mdx* EDL muscle following ECC with varying mechanical parameters. (A) Maximal tetanic rate of contraction for each protocol and (B) when grouped by length change. (C) Maximal tetanic rate of relaxation for each protocol and (D) when grouped by length change as a percent of initial following 10 eccentric contractions at 120 Hz using various protocols. ^*^ Different from the first protocol within a given length, ^#^ different from the second protocol within a given length, ^$^ different from the third protocol within a given length. Data are mean ± S.E.M with significance set at *p* < 0.05. *N* = 5 – 6/protocol.

**Additional file 3: Figure S3.** Ex vivo and in vivo force/torque frequency curve for *mdx* muscle. (A) The frequencies used in the ex vivo study of the EDL were 0, 35 and 120 Hz, which represent muscle lengthening without stimulation, that which elicited force half-way between a twitch and maximal tetanus, and the frequency required to generate a maximal tetanic contraction (381 ± 4 mN), respectively. *N* = 4. (B) Torque-frequency analysis of the anterior crural muscles using a 40° angle change at 2000°/s. Stimulation frequencies were 0, 52, 71, 93 and 150 Hz which represent ankle rotation without stimulation, and frequencies required to generate 50, 75, 90 and 100% of the difference between a twitch (1.04 ± 0.04 mN·m) and tetanus (2.84 ± 0.1 mN·m). *N* = 8.

**Additional file 4: Figure S4.** Isolated *mdx* EDL muscle does not lose isometric tetanic force following 10 passive lengthening manoeuvres. (A) Isometric tetanic force as a percent of initial following the 10th lengthening manoeuvre of various protocols at 0 Hz. (B) Isometric tetanic force as a percent of initial following the 10th eccentric contraction of various protocols at 0 Hz when collapsed into length changes. ^*^ Different from initial. Data are mean ± S.E.M with significance set at *p* < 0.05. *N* = 3/protocol.

**Additional file 5: Figure S5.** Submaximal ECC induce no to minimal loss of force in isolated *mdx* EDL muscle (A) Eccentric force (muscle tension) as a fraction of maximal isometric tetanic force (ECC:ISO force) for each eccentric protocol and (B) when collapsed into length changes. ^*^ Different from 5%, ^#^10%, ^$^20%. (C) Isometric tetanic force as a percent of initial for each protocol and (D) when collapsed into length changes following 10 eccentric contractions at 35 Hz. ^*^ Different from initial. Data are mean ± S.E.M with significance set at *p* < 0.05. *N* = 3/protocol.

**Additional file 6: Figure S6.** Ankle rotation impacts amount of work completed during ECC of *mdx* muscle in vivo. (A) Work completed by the anterior crural muscles during the first contraction for each eccentric protocol at 150 Hz and (B) when grouped by angle change. Statistics were only completed when grouped by angle change because three of the angle changes had an *n* = 1. ^*^ Different from 0°, ^#^5°, ^$^10°, ^Ɛ^20°. Data are mean ± S.E.M with significance set at *p* < 0.05. *N* = 3 – 6/protocol.

**Additional file 7: Figure S7.** Compound DS-11966966 increases maximal SERCA ATPase activity similar to CDN1163. After a 20-min incubation with compound, the Ca-ATPase activity of SERCA in SR vesicles isolated from skeletal muscle was measured at a calcium concentration (10 μM) that maximally activates SERCA, using an NADH-linked, enzyme-coupled activity assay [50]. ^*^ Different from0 μM compound (i.e., DMSO control). Data are mean ± S.E.M with significance set at *p* < 0.05. *N* = 5.

**Additional file 8: Figure S8.** SERCA1a activators and RyR1 leak inhibitors increase maximal rates of relaxation in isolated *mdx* muscle. (A) Maximal rates of tetanic relaxation in isolated EDL muscle of C57BL/10 and *mdx*. (B) Maximal rates of tetanic relaxation in the EDL muscle of *mdx* mice following the addition of 1% DMSO (vehicle; *p* = 0.460), (C) 1.0 μM DS-11966966, (D) 100 μM CDN1163, (E) 0.1 μM Chloroxine and (F) 100 μM Myricetin. ^*^ Different from C57BL/10, ^#^ different from *mdx*. Data are mean ± S.E.M with significance set at *p* < 0.05. *N* = 4 – 22/compound.

**Additional file 9: Figure S9.** Effects of SERCA1a activators and RyR1 leak inhibitors on maximal rates of contraction in isolated *mdx* muscle. (A) Maximal rates of tetanic contraction as a percent of tetanic plateau (maximal force of the EDL muscle attained prior to the 30 min incubation) in the EDL muscle of *mdx* mice following the addition of DS-11966966, (B) CDN1163, (C) Chloroxine and (D) Myricetin. *P* values represents One-way ANOVA and ^*^ different from vehicle at *p* < 0.05. Data are mean ± S.E.M. *N* = 3 – 9/ compound.

**Additional file 10: Figure S10.** SERCA1a and RyR1 small-molecule modulator concentrations on ECC force loss in *mdx* EDL muscle. Eccentric force loss induced by 5% length changes of isolated *mdx* EDL muscle incubated with SERCA1a activators (A) DS-11966966 and (B) CDN1163 or RyR1 leak inhibitors (C) Chloroxine and (D) Myricetin. *N* = 3 – 9/ compound.

## Data Availability

The datasets used and/or analyzed during the current study are available from the corresponding author on reasonable request.

## Abbreviations

+ dP/dt: Maximal rate of tetanic contraction
DMD: Duchenne muscular dystrophy
DMSO: Dimethyl sulfoxide
− dP/dt: Maximal rate of tetanic relaxation
EBD: Evan’s blue dye
ECC: Eccentric contraction
ECC:ISO: Ratio of maximal eccentric to isometric force
EDL: Extensor digitorum longus
*L*_o_: Optimal length of the muscle
NAC: N-acetylcysteine
*P*_o_: Maximal isometric tetanic force
ROS: Reactive oxygen species
RyR: Ryanodine receptor
SERCA: Sarco-endoplasmic reticulum calcium ATPase
SR: Sarcoplasmic reticulum
WT: Wildtype
