# Supplementary figures and images for: Mechanical factors tune the sensitivity of mdx muscle to eccentric strength loss and its protection by antioxidant and calcium modulators
Source: Skelet Muscle. 2020 Feb 1;10:3. doi: 10.1186/s13395-020-0221-2 (PMC6995146; doi:10.1186/s13395-020-0221-2)

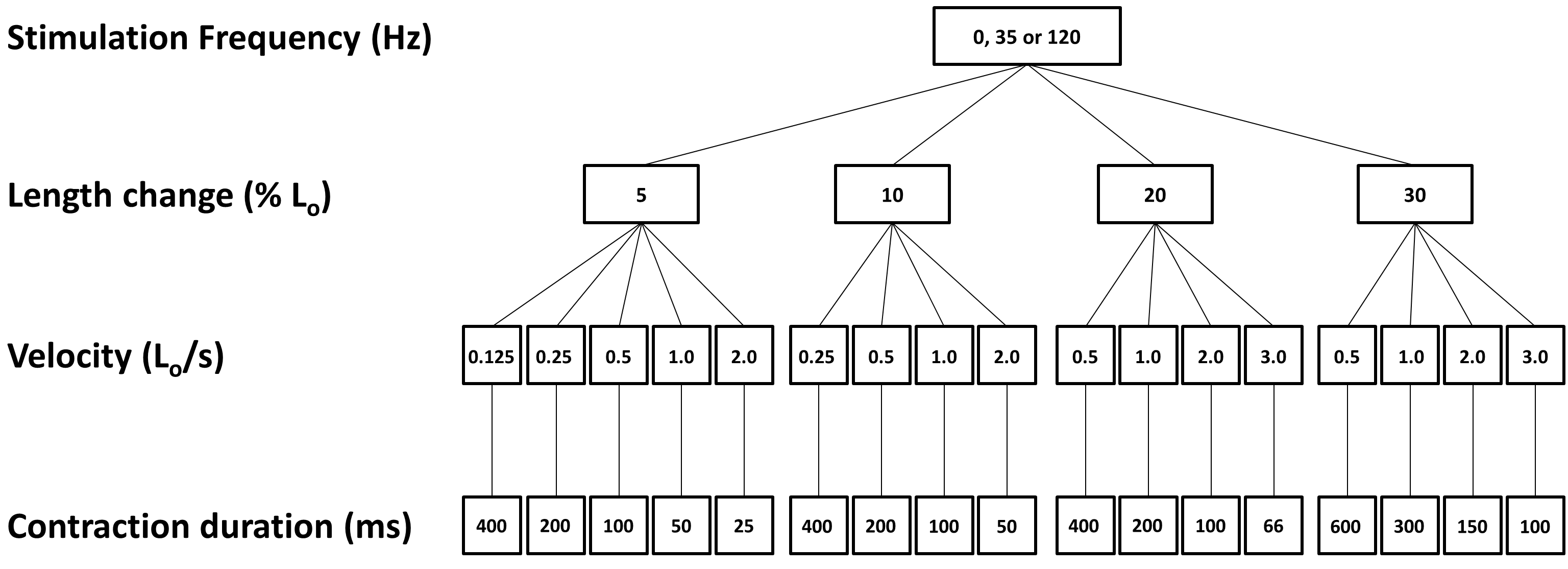

Supplement: Supplementary file 1 — Additional file 1: Figure S1. Summary of ex vivo eccentric contraction protocols used in this study. Lo = optimal muscle length. [file 13395_2020_221_MOESM1_ESM.tif]

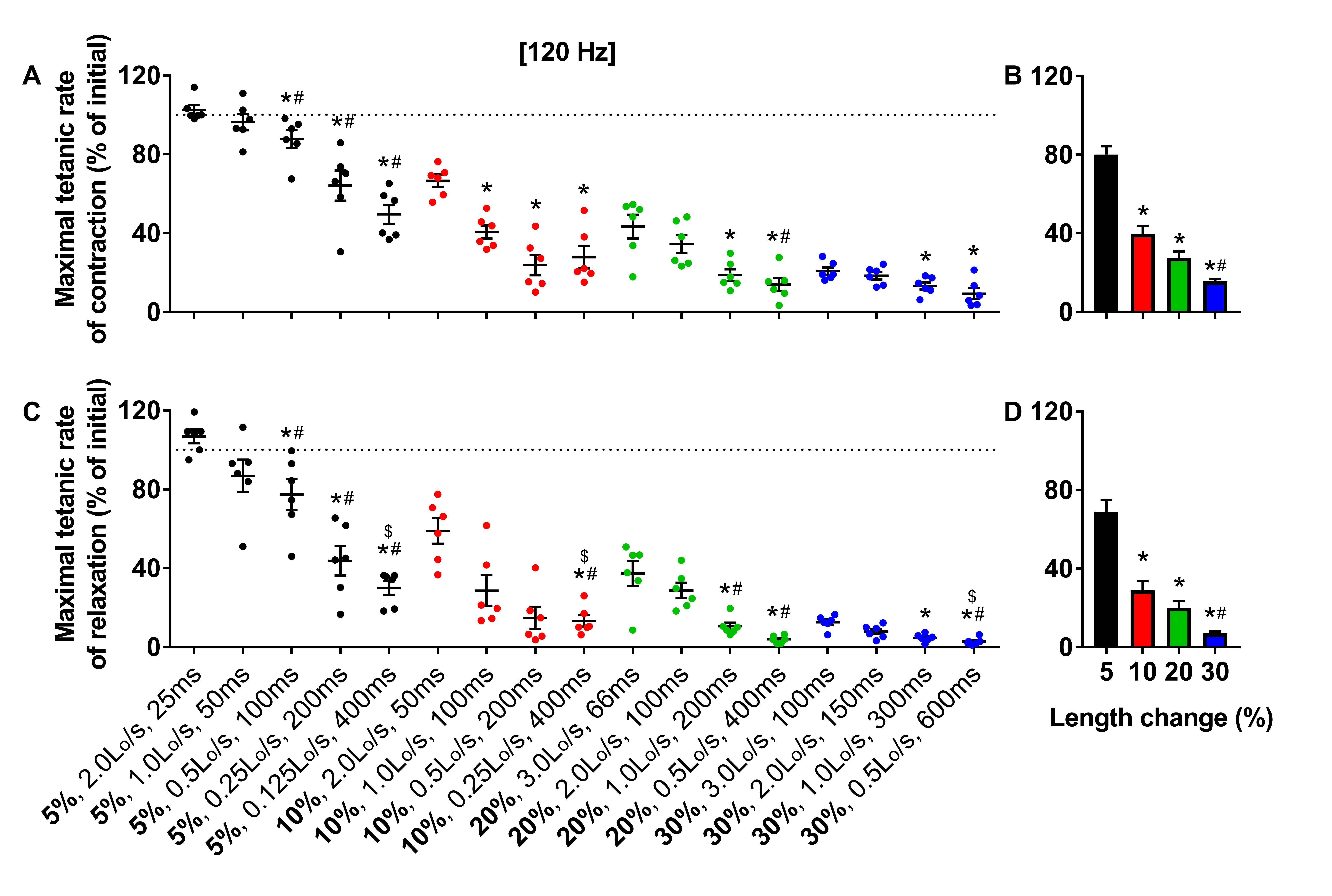

Supplement: Supplementary file 2 — Additional file 2: Figure S2. Rates of contraction and relaxation of mdx EDL muscle following ECC with varying mechanical parameters. (A) Maximal tetanic rate of contraction for each protocol and (B) when grouped by length change. (C) Maximal tetanic rate of relaxation for each protocol and (D) when grouped by length change as a percent of initial following 10 eccentric contractions at 120 Hz using various protocols. * Different from the first protocol within a given length, # different from the second protocol within a given length, $ different from the third protocol within a given length. Data are mean ± S.E.M with significance set at p < 0.05. N = 5 – 6/protocol. [file 13395_2020_221_MOESM2_ESM.tif]

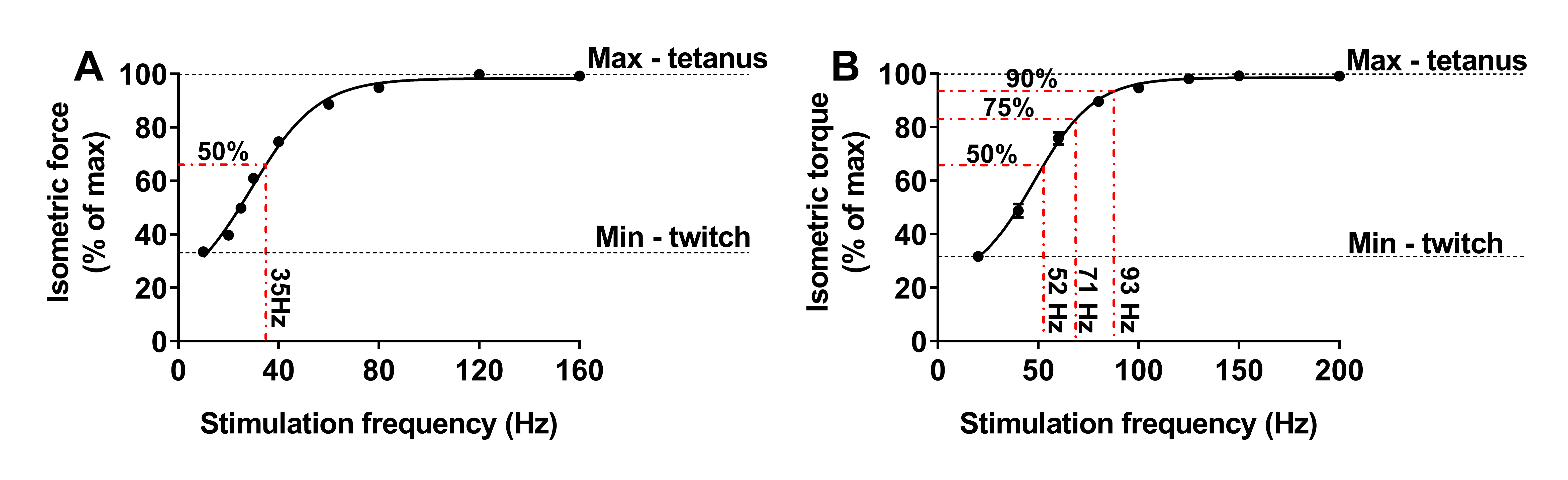

Supplement: Supplementary file 3 — Additional file 3: Figure S3. Ex vivo and in vivo force/torque frequency curve for mdx muscle. (A) The frequencies used in the ex vivo study of the EDL were 0, 35 and 120 Hz, which represent muscle lengthening without stimulation, that which elicited force half-way between a twitch and maximal tetanus, and the frequency required to generate a maximal tetanic contraction (381 ± 4 mN), respectively. N = 4. (B) Torque-frequency analysis of the anterior crural muscles using a 40° angle change at 2000°/s. Stimulation frequencies were 0, 52, 71, 93 and 150 Hz which represent ankle rotation without stimulation, and frequencies required to generate 50, 75, 90 and 100% of the difference between a twitch (1.04 ± 0.04 mN·m) and tetanus (2.84 ± 0.1 mN·m). N = 8. [file 13395_2020_221_MOESM3_ESM.tif]

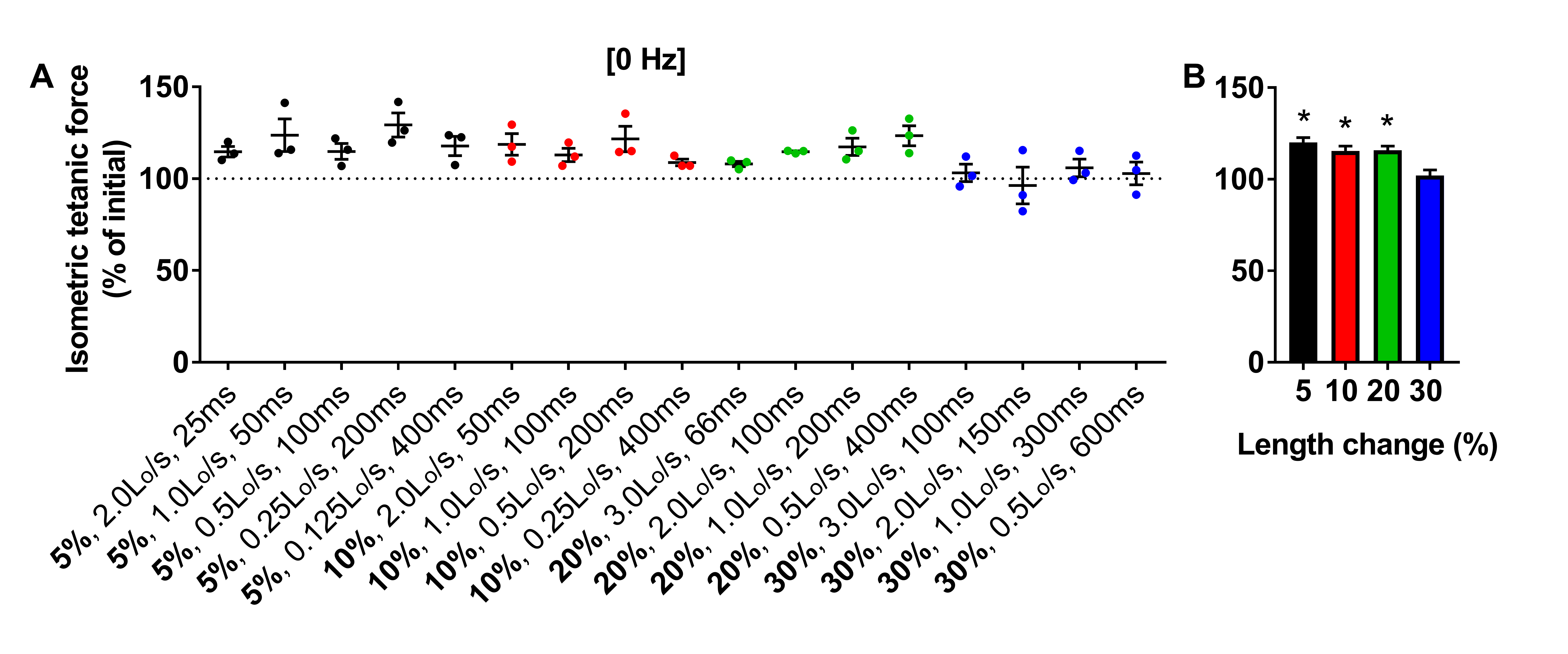

Supplement: Supplementary file 4 — Additional file 4: Figure S4. Isolated mdx EDL muscle does not lose isometric tetanic force following 10 passive lengthening manoeuvres. (A) Isometric tetanic force as a percent of initial following the 10th lengthening manoeuvre of various protocols at 0 Hz. (B) Isometric tetanic force as a percent of initial following the 10th eccentric contraction of various protocols at 0 Hz when collapsed into length changes. * Different from initial. Data are mean ± S.E.M with significance set at p < 0.05. N = 3/protocol. [file 13395_2020_221_MOESM4_ESM.tif]

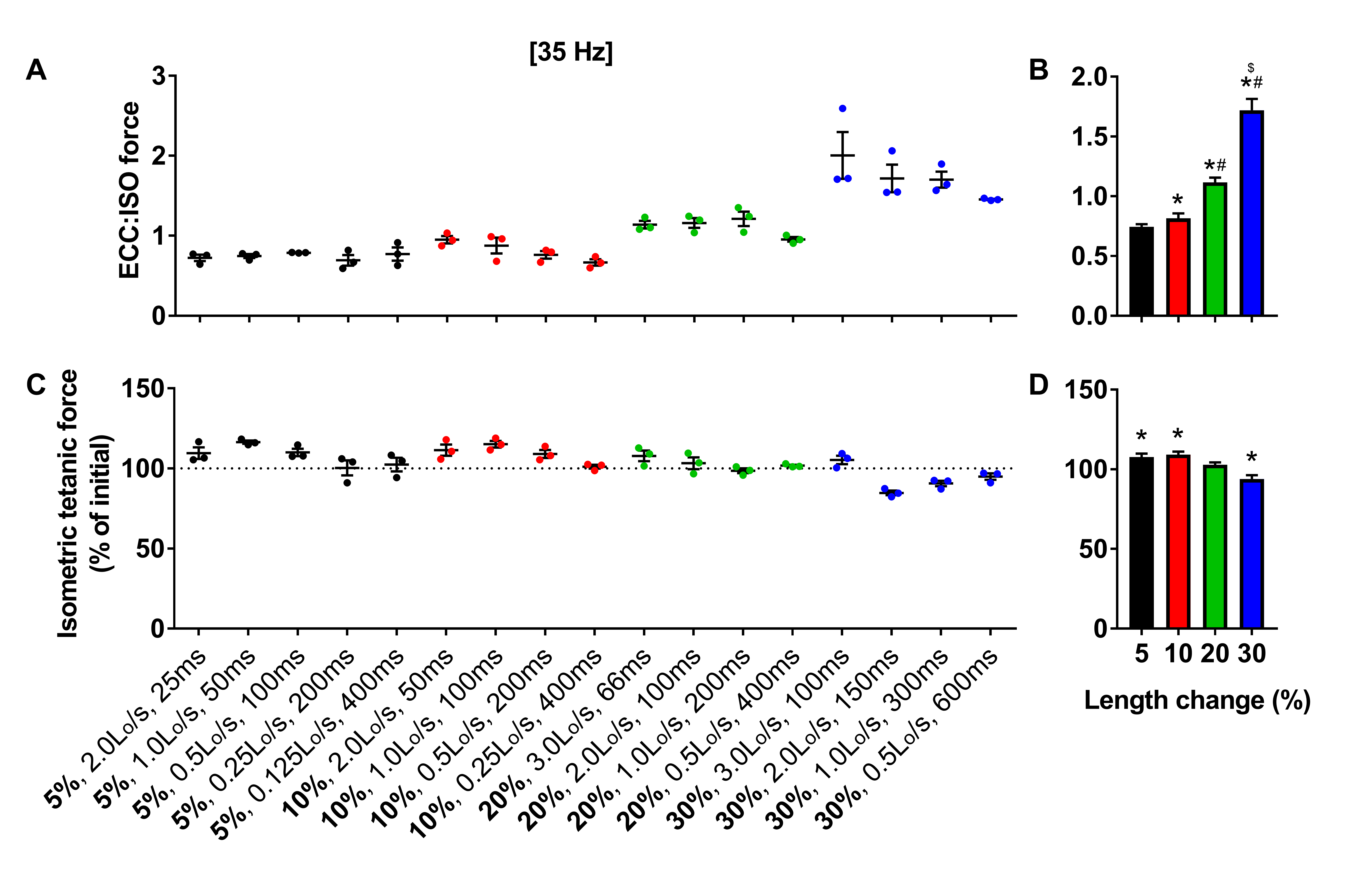

Supplement: Supplementary file 5 — Additional file 5: Figure S5. Submaximal ECC induce no to minimal loss of force in isolated mdx EDL muscle (A) Eccentric force (muscle tension) as a fraction of maximal isometric tetanic force (ECC:ISO force) for each eccentric protocol and (B) when collapsed into length changes. * Different from 5%, #10%, $20%. (C) Isometric tetanic force as a percent of initial for each protocol and (D) when collapsed into length changes following 10 eccentric contractions at 35 Hz. * Different from initial. Data are mean ± S.E.M with significance set at p < 0.05. N = 3/protocol. [file 13395_2020_221_MOESM5_ESM.tif]

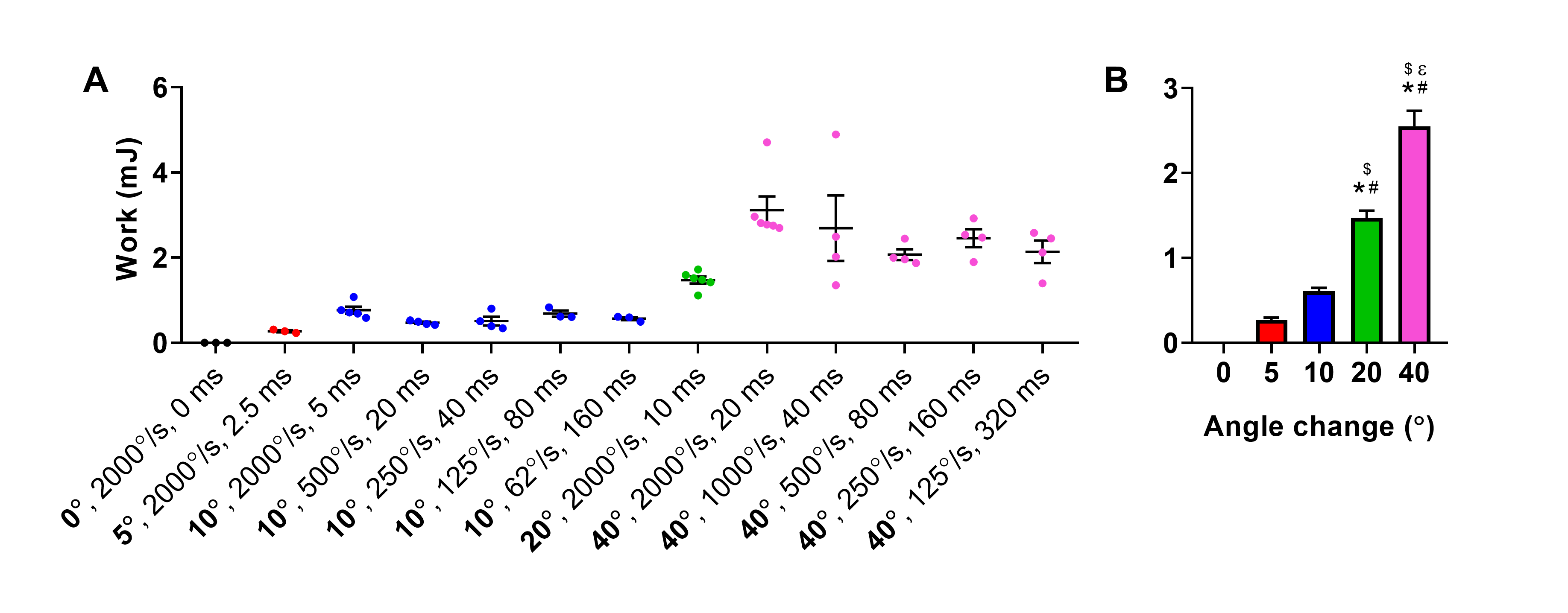

Supplement: Supplementary file 6 — Additional file 6: Figure S6. Ankle rotation impacts amount of work completed during ECC of mdx muscle in vivo. (A) Work completed by the anterior crural muscles during the first contraction for each eccentric protocol at 150 Hz and (B) when grouped by angle change. Statistics were only completed when grouped by angle change because three of the angle changes had an n = 1. * Different from 0°, #5°, $10°, Ɛ20°. Data are mean ± S.E.M with significance set at p < 0.05. N = 3 – 6/protocol. [file 13395_2020_221_MOESM6_ESM.tif]

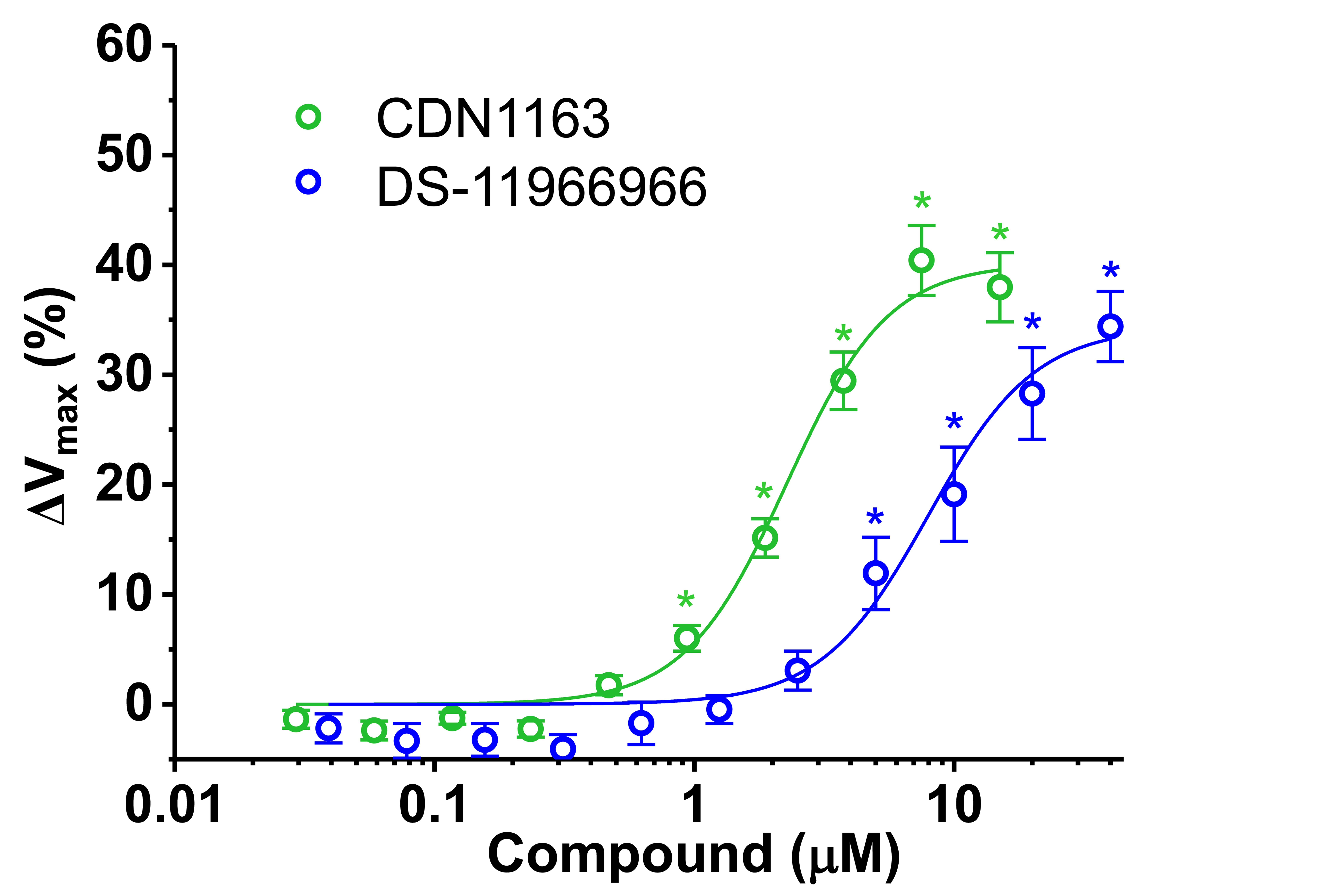

Supplement: Supplementary file 7 — Additional file 7: Figure S7. Compound DS-11966966 increases maximal SERCA ATPase activity similar to CDN1163. After a 20-min incubation with compound, the Ca-ATPase activity of SERCA in SR vesicles isolated from skeletal muscle was measured at a calcium concentration (10 μM) that maximally activates SERCA, using an NADH-linked, enzyme-coupled activity assay [50]. * Different from0 μM compound (i.e., DMSO control). Data are mean ± S.E.M with significance set at p < 0.05. N = 5. [file 13395_2020_221_MOESM7_ESM.tif]

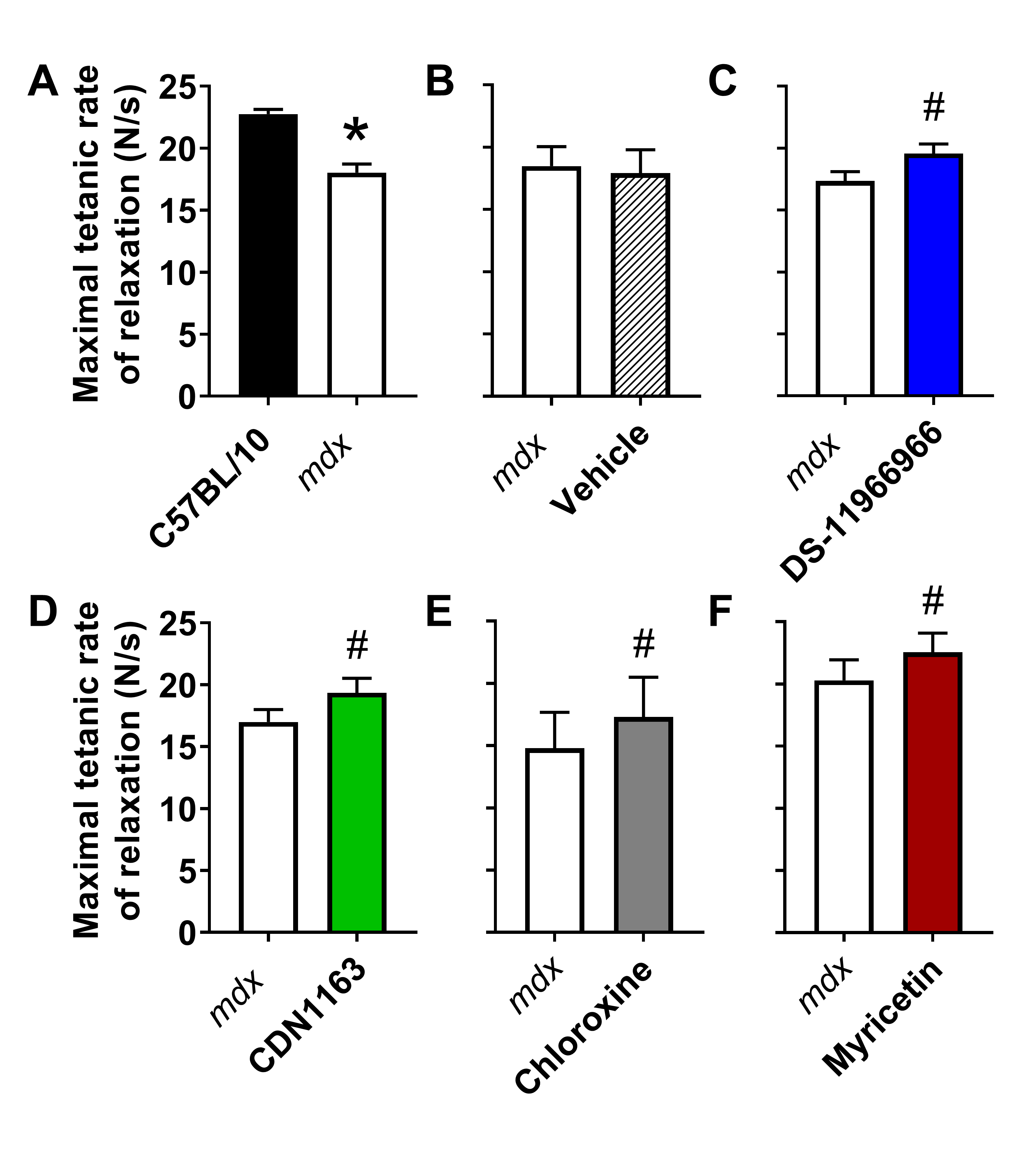

Supplement: Supplementary file 8 — Additional file 8: Figure S8. SERCA1a activators and RyR1 leak inhibitors increase maximal rates of relaxation in isolated mdx muscle. (A) Maximal rates of tetanic relaxation in isolated EDL muscle of C57BL/10 and mdx. (B) Maximal rates of tetanic relaxation in the EDL muscle of mdx mice following the addition of 1% DMSO (vehicle; p = 0.460), (C) 1.0 μM DS-11966966, (D) 100 μM CDN1163, (E) 0.1 μM Chloroxine and (F) 100 μM Myricetin. * Different from C57BL/10, # different from mdx. Data are mean ± S.E.M with significance set at p < 0.05. N = 4 – 22/compound. [file 13395_2020_221_MOESM8_ESM.tif]

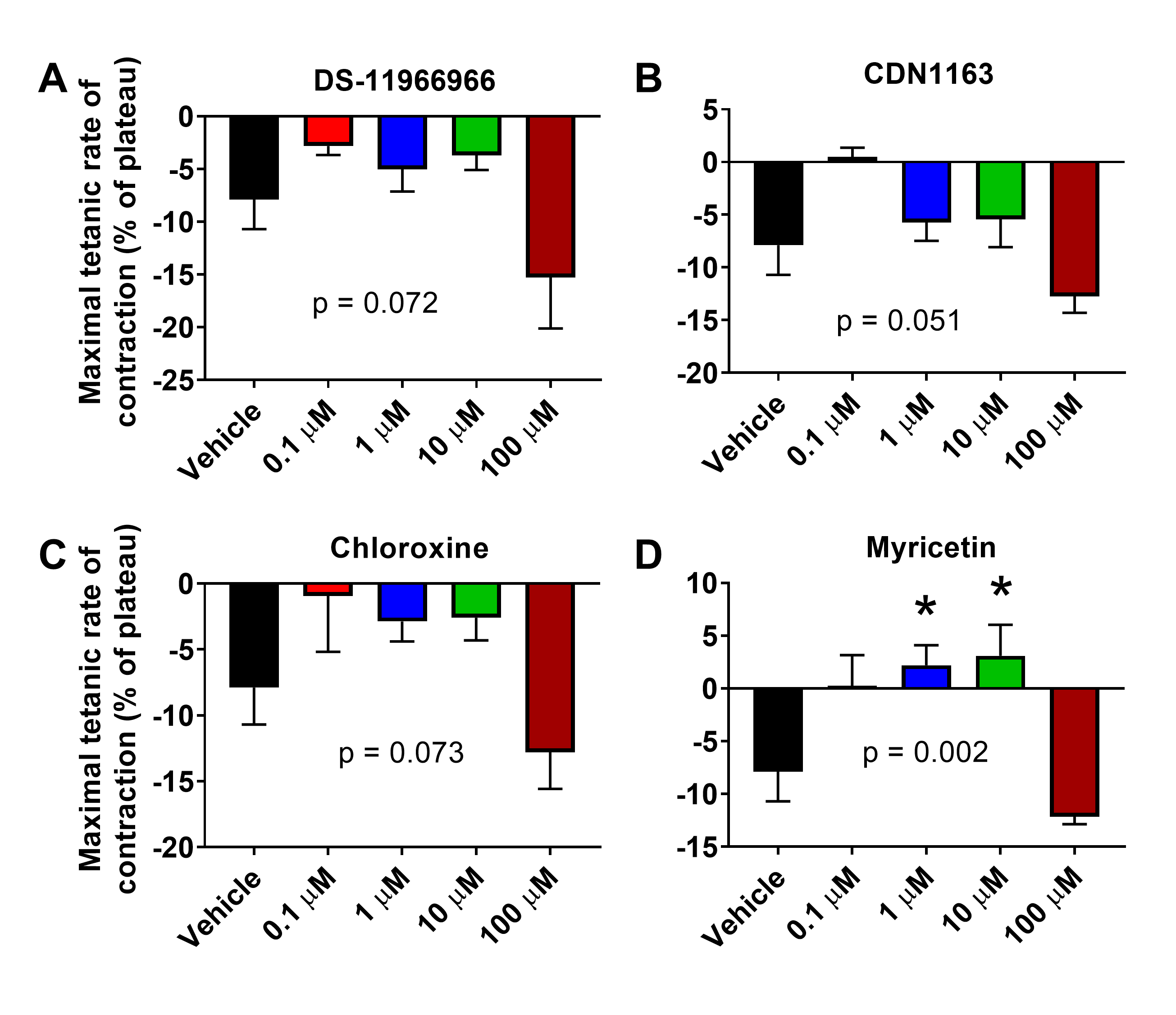

Supplement: Supplementary file 9 — Additional file 9: Figure S9. Effects of SERCA1a activators and RyR1 leak inhibitors on maximal rates of contraction in isolated mdx muscle. (A) Maximal rates of tetanic contraction as a percent of tetanic plateau (maximal force of the EDL muscle attained prior to the 30 min incubation) in the EDL muscle of mdx mice following the addition of DS-11966966, (B) CDN1163, (C) Chloroxine and (D) Myricetin. P values represents One-way ANOVA and * different from vehicle at p < 0.05. Data are mean ± S.E.M. N = 3 – 9/ compound. [file 13395_2020_221_MOESM9_ESM.tif]

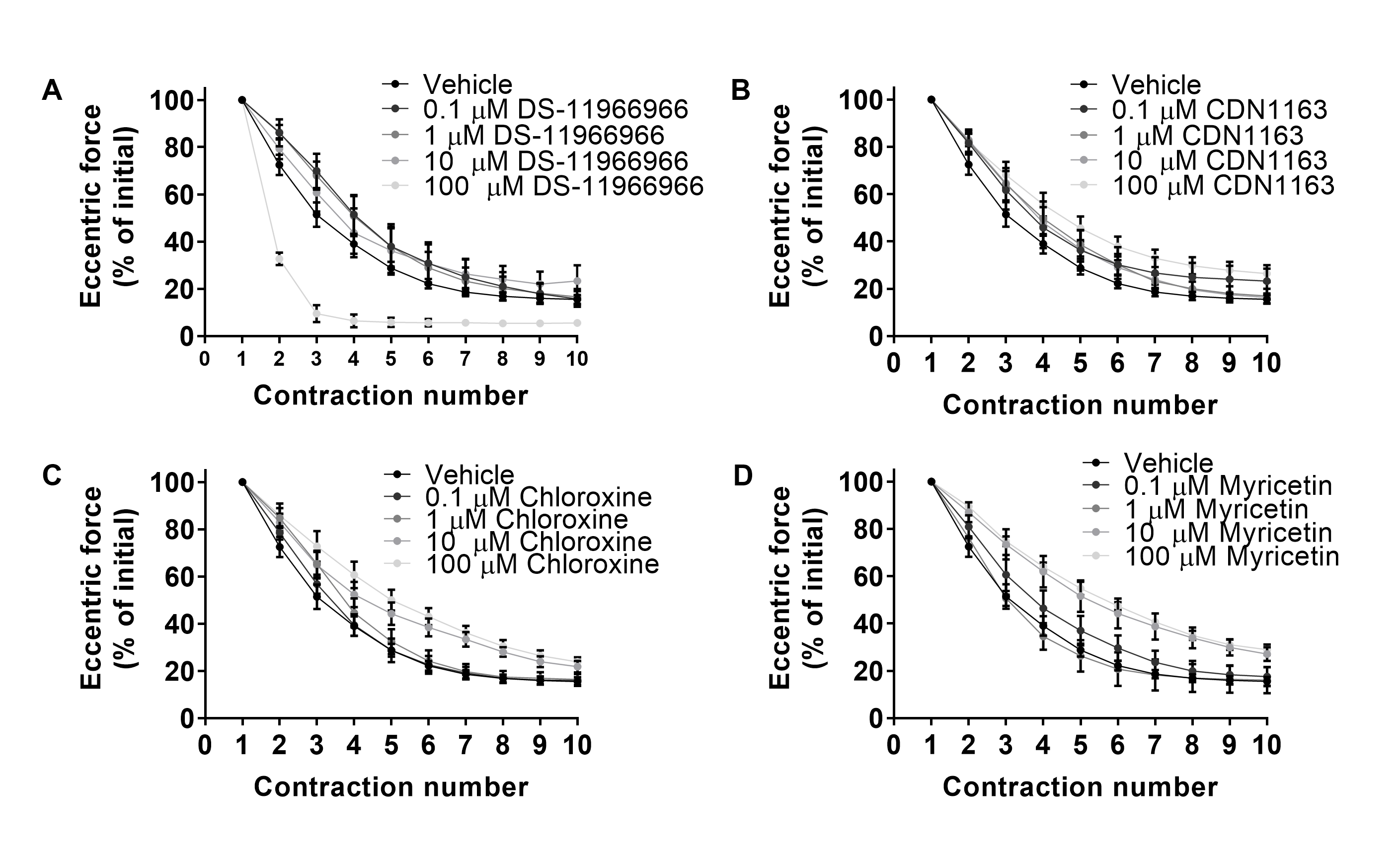

Supplement: Supplementary file 10 — Additional file 10: Figure S10. SERCA1a and RyR1 small-molecule modulator concentrations on ECC force loss in mdx EDL muscle. Eccentric force loss induced by 5% length changes of isolated mdx EDL muscle incubated with SERCA1a activators (A) DS-11966966 and (B) CDN1163 or RyR1 leak inhibitors (C) Chloroxine and (D) Myricetin. N = 3 – 9/ compound. [file 13395_2020_221_MOESM10_ESM.tif]
